# Transcriptomic network analyses of leaf dehydration responses identify highly connected ABA and ethylene signaling hubs in three grapevine species differing in drought tolerance

**DOI:** 10.1186/s12870-016-0804-6

**Published:** 2016-05-23

**Authors:** Daniel W. Hopper, Ryan Ghan, Karen A. Schlauch, Grant R. Cramer

**Affiliations:** Department of Biochemistry and Molecular Biology, University of Nevada, Reno, NV 89557 USA

**Keywords:** ABA, ABI5, Dehydration, Ethylene, Grapevine, Network analysis, Transcriptomics, *Vitis*, WGCNA

## Abstract

**Background:**

Grapevine is a major food crop that is affected by global climate change. Consistent with field studies, dehydration assays of grapevine leaves can reveal valuable information of the plant’s response at physiological, transcript, and protein levels. There are well-known differences in grapevine rootstocks responses to dehydration. We used time-series transcriptomic approaches combined with network analyses to elucidate and identify important physiological processes and network hubs that responded to dehydration in three different grapevine species differing in their drought tolerance.

**Results:**

Transcriptomic analyses of the leaves of Cabernet Sauvignon, Riparia Gloire, and Ramsey were evaluated at different times during a 24-h controlled dehydration. Analysis of variance (ANOVA) revealed that approximately 11,000 transcripts changed significantly with respect to the genotype x treatment interaction term and approximately 6000 transcripts changed significantly according to the genotype x treatment x time interaction term indicating massive differential changes in gene expression over time. Standard analyses determined substantial effects on the transcript abundance of genes involved in the metabolism and signaling of two known plant stress hormones, abscisic acid (ABA) and ethylene. ABA and ethylene signaling maps were constructed and revealed specific changes in transcript abundance that were associated with the known drought tolerance of the genotypes including genes such as *VviABI5*, *VviABF2*, *VviACS2*, and *VviWRKY22*. Weighted-gene coexpression network analysis (WGCNA) confirmed these results. In particular, WGCNA identified 30 different modules, some of which had highly enriched gene ontology (GO) categories for photosynthesis, phenylpropanoid metabolism, ABA and ethylene signaling. The ABA signaling transcription factors, *VviABI5* and *VviABF2,* were highly connected hubs in two modules, one being enriched in gaseous transport and the other in ethylene signaling. *VviABI5* was distinctly correlated with an early response and high expression for the drought tolerant Ramsey and with little response from the drought sensitive Riparia Gloire. These ABA signaling transcription factors were highly connected to *VviSnRK1* and other gene hubs associated with sugar, ethylene and ABA signaling.

**Conclusion:**

A leaf dehydration assay provided transcriptomic evidence for differential leaf responses to dehydration between genotypes differing in their drought tolerance. WGCNA proved to be a powerful network analysis approach; it identified 30 distinct modules (networks) with highly enriched GO categories and enabled the identification of gene hubs in these modules. Some of these genes were highly connected hubs in both the ABA and ethylene signaling pathways, supporting the hypothesis that there is substantial crosstalk between the two hormone pathways. This study identifies solid gene candidates for future investigations of drought tolerance in grapevine.

**Electronic supplementary material:**

The online version of this article (doi:10.1186/s12870-016-0804-6) contains supplementary material, which is available to authorized users.

## Background

Boyer [1] reviewed the impact of the environment on crop production and highlighted the need for crops better suited to these environments. Much has been learned since then, yet our understanding of plant responses to abiotic and biotic stresses is very incomplete. Drought signaling within plants is a complex process involving many different signaling cascades [2]. A rapid assay was developed to assess the physiological response of different grapevine genotypes to dehydration [3]. This assay is a simple approach that can determine differences in dehydration sensitivity at the physiological and Omic levels.

Climate change is expected to affect water and land availability [4, 5]. Rootstocks are used in viticulture because they can confer pest or drought resistance, alter vigor to the scion or the fruit-bearing portion of the plant, thus, impacting fruit quality; rootstocks are vital in most viticultural regions [6, 7]. Much research has focused on the scion-rootstock relationship [8–11], but there is little research on the rootstock response to abiotic conditions.

Three different *Vitis* genotypes were shown previously to have differences in their dehydration sensitivity [3, 6, 12]. Cabernet Sauvignon (*Vitis vinifera* L.) along with two North American *Vitis* species commonly used as rootstocks, Ramsey (*Vitis champinii* Planch., a naturally occurring hybrid between *Vitis candicans* Engelm*.* and *Vitis rupestris* Scheele) and Riparia Gloire (*Vitis riparia* Michx.). Ramsey, a drought tolerant genotype, originates from hot, dry regions of Texas. Riparia Gloire originates from wet, riparian areas and is drought sensitive [3, 6, 12].

Transcriptomic analyses allow one to have a “holistic” snapshot of the plant’s transcriptional response to a changing environment [2]. A time-series transcriptomic analysis allows one to begin to elucidate the sensitivities of the response and the primary or secondary responses. Co-expression analyses allow one to identify networks, genes that have high connectivity or correlation with each other. A particularly powerful approach is the weighted coexpression network analysis (WGCNA) method [13–16]. This analysis can identify clusters (modules) of genes with high biological meaning. It can also identify those genes with high connectivity or module membership, which are essentially hub genes. Similar to airport hubs, if one hub is not functioning, the whole system can slow down or become chaotic. Therefore we consider hub genes important or essential genes for the proper functioning of the system or organism.

Abscisic acid (ABA) and ethylene are two important hormones that regulate abiotic stress responses in plants [2, 17]. In a preliminary survey of more than 30 genotypes, we found variation in the increase in transcript abundance of *VviNCED3*, the rate-limiting step in ABA biosynthesis, in response to rapid dehydration. We hypothesize that the transcriptomic responses to rapid dehydration between these grapevine genotypes are different and may involve ABA signaling. In this time-series transcriptomic study, we identify significant genes by standard and network (WGCNA) analysis methods; a number of these genes are involved in ABA and ethylene signaling and correlate with the relative drought tolerance between the genotypes. In particular, *ABI5*, a transcription factor gene normally associated with ABA regulation of germination, was highly sensitive and increased in Ramsey leaves, the most drought tolerant of the three genotypes, early in the response to dehydration, but there was little effect of dehydration on abscisic acid insensitive 5 (ABI5) transcription factor expression in Riparia Gloire leaves, the most drought sensitive of the genotypes. This gene and other hub genes are identified as “solid” candidates for future drought tolerance research.

## Methods

### Plant material and experimental conditions

Three grapevine genotypes (*Vitis vinifera* cv. Cabernet Sauvignon clone 8, *Vitis riparia* cv. Riparia Gloire, and *Vitis champinii* cv. Ramsey (a naturally occurring hybrid between *Vitis candicans* and *Vitis rupestris*)) were pruned to two shoots and grown in 13.3 liter pots containing 10 L SuperSoil® potting mix supplemented with slow release fertilizer (5-10-10). The original vine cuttings of Riparia Gloire and Ramsey were obtained from Dr. Andrew Walker at the University of California, Davis, CA, USA. The original vine cuttings of Cabernet Sauvignon vines were obtained from Inland Desert Nursery in Benton City, WA, USA. Vines were grown in a greenhouse with supplemental sodium vapor lamp lighting (day/night cycles of 16 h/8 h light (minimum 400 μE m^−2^ s^−1^) and 28 °C/18 °C). Fully-developed leaves were subjected to dehydration as previously described [3]. Leaves were removed from dehydration boxes at 1, 2, 4, 8, and 24 h and frozen immediately in liquid nitrogen. Control leaves were taken from the second shoot of the intact plant at the corresponding daytime of the dehydration assay to account for circadian effects on transcript abundance.

### RNA extraction and microarray hybridization

Frozen leaves were ground using a Retsch MM 301 ball mill [18] for 1 min at 30 revolutions s^−1^. Total RNA was extracted from approximately 100 mg of tissue using a cetyl trimethylammonium bromide (CTAB)-based method [19, 20]. Extracts were treated with DNase (Qiagen RNeasy Plant Kit, [21]) according to manufacturer’s instructions. RNA quality and quantity were assessed with a Nanodrop ND-1000 spectrophotometer (ThermoFisher Scientific, Waltham, MA) and an Agilent 2100 Bioanalyzer (Agilent Technologies, Santa Clara, CA, USA) according to the manufacturer’s instructions. Microarrays were hybridized by MOgene (St. Louis, MO, USA) using the NimbleGen microarray 090818 Vitis exp HX12 (Roche, NimbleGen Inc., Madison, WI, USA) according to the manufacturer’s instructions.

### Statistical analysis

All microarrays were analyzed as one set as previously described [22–24]. One of ninety arrays (Rip4S2) exhibited considerable spatial variation, and thus was excluded. Two other arrays (Cab4S3 and Cab8S1) had notable statistically significant outliers across replicates when compared to other arrays, and were thus also excluded.

A simple 3-way analysis of variance (ANOVA) was performed on normalized (log-transformed) and quality-controlled processed data to determine which probesets on the array were differentially expressed with statistical significance across Genotype, Treatment, and Time, and the 2-way and 3-way interaction of these effects. The processed and normalized expression values were not normally distributed, thus an extension of the Kruskal Wallis rank sum test was used for the ANOVA [25]. A multiple testing correction was applied to the *p*-values of the ANOVA [26], and any probeset with a significant Genotype **x** Treatment or Genotype **x** Treatment **x** Time interaction term with adjusted *p*-value ≤ 0.05 was examined further.

Principal component analysis (PCA) was applied to quality-controlled expression data using the covariance matrix to visualize any trends in the expression data [27–30]. The PCA (Additional file 1) showed a very clear separation between genotypes, supporting the large number of probesets with a statistically significant tissue effect (95.6 %).

Gene expression was also evaluated with WGCNA [15] using the following settings for the adjacency function (datExpr, power = 16, type = "signed hybrid", corFnc = "bicor", corOptions = "use = 'p', maxPOutliers = 0.1") and for the cuttreeDynamic function (dendro = geneTree, distM = dissTOM, method = "hybrid", deepSplit = 2, pamRespectsDendro = F, minClusterSize = 30); these functions have been shown to be the best approach for biologically meaningful results [16]. WGCNA also confirmed clear separation by genotype and treatment (Additional file 2).

Functional categorization of significant transcripts was performed with the BiNGO plugin [31] in Cytoscape [32] using a gene ontology (GO) file created with the EnsemblPlants BioMart [33] for *Vitis vinifera*. Overrepresented (enriched) categories were determined using a hypergeometric test with a significance threshold at 0.05 after a Benjamini and Hochberg false discovery rate correction.

## Results

### Dehydration causes massive changes in gene expression

Fully mature Cabernet Sauvignon, Riparia Gloire, and Ramsey leaves were dehydrated [3] to asses rapid transcriptomic changes. Briefly, the leaf was excised from the plant and placed into a dehydration box for various time points over a 24-h period. Leaf dehydration occurred in the air above a solution of NaCl in a sealed container in a growth chamber. Leaves were removed from the box and immediately frozen in liquid nitrogen at specific time points of dehydration. Control samples were taken from the same plant at the corresponding time to account for any circadian effect on transcript abundance. RNA was extracted from three experimental replicates for treatment and control samples at each time point.

In order to test the hypothesis that the response to rapid dehydration between the different genotypes is different over time at the transcript level, a 2 × 2 × 5 factorial (Genotype **x** Treatment **x** Time) experimental design was established. Transcriptomic analysis was carried out using the NimbleGen Grape Whole-Genome Microarray. A parametric ANOVA was originally performed but because the expression data were not quite normally distributed, the expression data were reanalyzed using a nonparametric ANOVA. The nonparametric ANOVA reduced the overall number of genes with differential expression by about 10 %. The nonparametric ANOVA determined that the abundance of 28,030 transcripts changed significantly with an adjusted *p*-value ≤ 0.05 (herein referred to as “significant” throughout the paper) with respect to genotype (Table 1, Additional file 3). Clearly there are large differences in gene expression between these species regardless of treatment. There were 18,237 and 23,656 transcripts that changed significantly with Treatment and Time, respectively; 11,436 transcripts changed significantly with respect to Genotype **x** Treatment; 24,543 transcripts changed significantly with genotype over time; 17,488 transcripts changed significantly with Treatment **x** Time, and finally 6,285 transcripts changed significantly for the Genotype **x** Treatment **x** Time term (Table 1, Additional file 3).

**Table 1:** Number of significant transcripts for each effects and interaction term in the ANOVA. Significance mentioned in the manuscript refers to an adjusted *p* ≤ 0.05

Genotype (G)	Treatment (TRT)	Time	G x Trt	G x Time	Trt x Time	G x Trt x Time
28,030	18,237	23,656	11,436	24,543	17,488	6285

Two approaches were taken to analyze these large datasets: “standard analysis” and “network analysis”. With standard analysis we used *a posteriori* knowledge to sort through known biochemical and signaling pathways affected by dehydration. With network analysis, we took an *a priori* approach by using WGCNA and GO enrichment methods.

As we are interested in elucidating mechanisms of drought tolerance, we focused on the Genotype x Treatment and the Genotype x Treatment x Time interaction sets of genes. GO categories for these gene sets were determined with a custom *Vitis* GO file (see Methods) and analyzed for significant overrepresentation using BiNGO, a Cytoscape plug-in [31]. However, GO enrichment analysis was not very informative with these large datasets. There were two biological processes significantly overrepresented for the Genotype **x** Treatment gene set (Additional file 4): translation and phenylpropanoid metabolism (more will be discussed about these categories later in the network analysis section). The Genotype **x** Treatment **x** Time gene set had one category that was significantly overrepresented: oxidation-reduction (Additional file 5). This latter interaction term did not provide any obvious clues about differences between the genotypes, so we used previous knowledge from our research to ascertain if there were differences in ABA metabolism and signaling, the hypothesis in which we were most interested.

### Dehydration induces significant changes in ABA metabolism transcripts between the genotypes

**Fig. 1:**
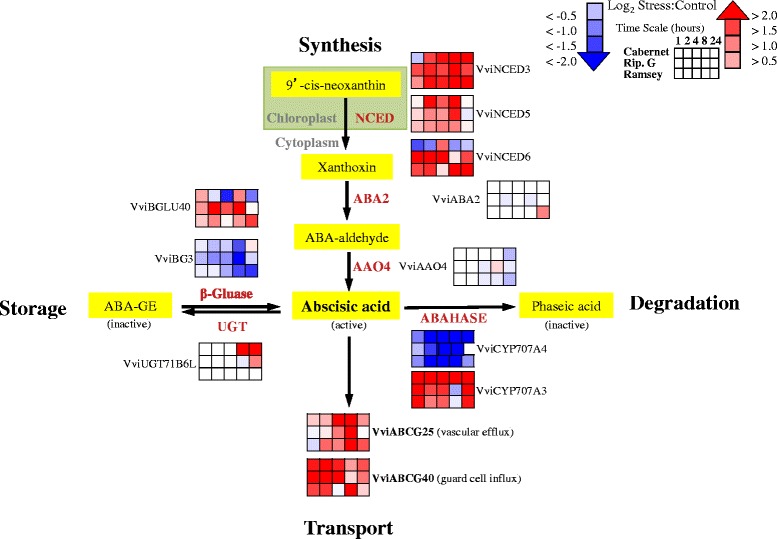
A simplified model of transcripts involved in ABA metabolism and transport. Corresponding *Vitis* loci ID and ANOVA results for the gene symbols used in this figure are listed in Additional file 6. Data are presented as heatmaps of mean values of a log_2_ ratio (Stress:Control), *n* = 3 at each time point

The rate-limiting step in ABA biosynthesis is catalyzed by genes that encode 9-cis-epoxycarotenoid dioxygenase (NCED) [34, 35]. In *Vitis* there are three NCED genes, which can lead to the production of ABA [36]. The gene symbols used are based upon the symbol used to the closest ortholog in *Arabidopsis. Vitis* and *Arabidopsis* loci for these symbols are listed in Additional file 6. A significant difference in the transcript abundance for these three genes was observed (Fig. 1). In Cabernet Sauvignon, *VviNCED3* expression was slightly decreased at 1 h of dehydration while the expression in Riparia Gloire and Ramsey was increased in response to dehydration. Riparia Gloire had larger initial response than Ramsey, but after 4 h, *VviNCED3* expression of Ramsey and Cabernet Sauvignon exceeded that of Riparia Gloire. These results confirmed our preliminary results that there were differences between the genotypes in the expression of *VviNCED3* in response to dehydration.

*VviNCED5* transcript abundance increased in Cabernet Sauvignon leaves from 2 to 8 h; there was an increase in Riparia Gloire at the 8 h time point and a slight increase in Ramsey (Fig. 1). The transcript abundance of *VviNCED6* in both North American genotypes increased within 1 h of dehydration, but there was little response in Cabernet Sauvignon (Fig. 1). These data indicate a different regulation of these genes in response to dehydration as well as differences between the genotypes.

ABA action within the plant is also dependent on degradation, conjugation and transport. Significant differences in the transcript abundance of genes involved in these processes were different between the genotypes. Degradation of ABA is catalyzed by a group of cytochrome P450 enzymes known as ABA-hydroxylases, and then continues by a few non-enzymatic steps leading to the formation of phaseic acid. Two genes annotated to be ABA-hydroxylases, *VviCYP707A3* and *VviCYP707A4*, responded differently to the dehydration (Fig. 1). The transcript abundance of *VviCYP707A4* decreased throughout the experiment for all genotypes. At 4 h of dehydration, transcript abundance in both Riparia Gloire and Ramsey were at their lowest points with a log_2_ fold decrease greater than 4. Interestingly, *VviCYP707A3* increased in expression throughout the experiment, most notably in Cabernet Sauvignon (Fig. 1).

Active ABA can also be produced through β-glucosidase, which involves the hydrolysis of an inactive form of ABA, Glc-conjugated ABA (ABA-GE), to active ABA. These enzymes are localized in the vacuole where ABA-GE is known to be stored [37]. Previously, Zhang et al. [38] found significant differences in the expression of three genes in *Vitis* encoding β-glucosidases in *Vitis vinifera* cv. Muscat Hamburg berry ripening. In our study, transcript abundance of *VviBGLU40* increased in Riparia Gloire leaves, most notably at 2 h of dehydration (Fig. 1). Interestingly, Zhang et al. [38] reported that *VviBG3* transcript abundance decreased through véraison in berry samples. A similar expression profile was observed in this study in all genotypes surveyed, most notably in Riparia Gloire at 8 h of dehydration (Fig. 1).

ABA transport can also affect ABA concentrations. The transcript abundance of two ABA transporters was shown to increase significantly in response to dehydration (Fig. 1) and was different between the genotypes over time. *VviABCG25* is an ATP-binding cassette (ABC) transporter that exports ABA from vascular tissues allowing ABA to reach distant guard cells [39, 40]. After 4 h of dehydration, transcript abundance increased with a log_2_ fold change of more than 2. All genotypes displayed a large increase in transcript abundance by 8 h of dehydration.

In *Arabidopsis,* AtABCG40 imports ABA directly into guard cells [41]. In our study, transcript abundance in all genotypes increased within 1 h of dehydration (Fig. 1). Interestingly, Riparia Gloire increased nearly 5-fold indicating a massive change in transcript abundance. This gene may contribute to the dehydration and ABA sensitivity of stomatal conductance of Riparia Gloire leaves [3].

Together these results indicate that transcripts involved in ABA metabolism changed significantly in response to rapid dehydration and the responses between the genotypes were different, consistent with the differences in dehydration sensitivity previously observed [3].

### ABA core-signaling response to dehydration

**Fig. 2:**
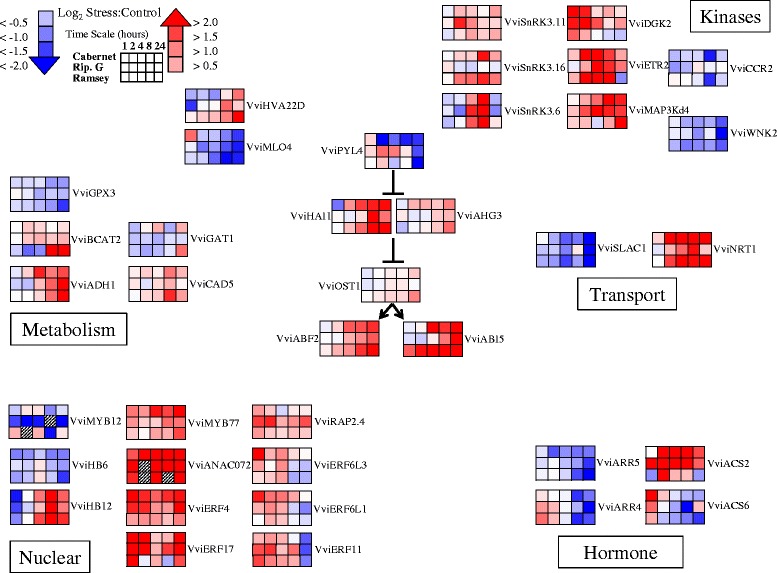
Representation of the transcript abundance of some of the genes of the ABA core interactome. Corresponding *Vitis* loci ID and ANOVA results for the gene symbols used in this figure are listed in Additional file 6. Data are presented as heatmaps of mean values of a log_2_ ratio (Stress:Control), *n* = 3 at each time point

Downstream of ABA biosynthesis is a complex ABA signaling network involving many different genes. Recently, Lumba et al. [42] took a systems biology approach to create an ABA core-signaling network consisting of over 500 interactions between 138 proteins in *Arabidopsis*. Many different processes are represented such as proteins involved in transport, metabolism, proteolysis, calcium sensing, as well as numerous transcription factors and kinases. *Vitis* orthologs were compiled based on the closest orthologs identified by Gramene ([43] release 44 (January 2015); see list in Additional file 6. Significant differences in gene expression within the ABA core-signaling network were detected (Fig. 2).

ABA binds to receptors in the cytoplasm known as PYR/PYL/RCAR proteins [44, 45]. Evidence indicates that there are additional receptors located at the plasma membrane [46]. Interestingly, the transcript abundance of *VviPYL4* changed significantly in response to dehydration between the genotypes; transcript abundance was decreased in Cabernet Sauvignon leaves while there was a slight increase in Riparia Gloire and little response in Ramsey (Fig. 2). ABA receptors interact with specific type 2C protein phosphatases (PP2C), which inhibit the activity of serine/threonine-protein kinase 2.6 (SnRK2.6; OST1) when ABA is not present. In the presence of ABA, PP2C proteins dissociate from the kinase allowing autophosphorylation and subsequent downstream signaling. The transcript abundance of two PP2C genes, *VviHAI1* and *VviAHG3*, increased significantly in response to dehydration between the genotypes (Fig. 2). The transcript abundance of *VviOST1* significantly increased in response to dehydration, most notably in Ramsey after 24 h of dehydration (Fig. 2). Downstream targets of *VviOST1* also increased significantly in response to dehydration (Fig. 2). Targets include an ABA-responsive binding elements factor (ABF) that contains an ABA-responsive element (ABRE; PyACGTGG/TC) as a conserved *cis-*element in the promoter region [47]. In our study, *VviABF2* transcript abundance increased significantly in response to dehydration in all the genotypes (Fig. 2). Another ABF protein that is a target of OST1 is ABI5. *VviABI5* transcript abundance increased significantly in response to dehydration, most notably in Ramsey leaves as early as 1 h of dehydration (Fig. 2). Recently, Yoshida et al. [48] noted that ABF2 in *Arabidopsis* is one of four predominant AREB/ABF transcription factors downstream of SnRK2.6 (OST1) in response to various osmotic stress conditions. Interestingly, the authors did not see a significant increase in the expression of *AtABI5* under their conditions. In our study, there is clear evidence for the induction and differential expression of *VviABI5* in response to dehydration.

A number of genes are induced by ABA, but lack the specific binding element mentioned above including proteins known to be involved in transport. The transcript abundance of two genes, guard cell S-type anion channel, *VviSLAC1,* and nitrate transporter, *VviNRT1*, changed significantly in response to dehydration (Fig. 2). SLAC1 is required for stomatal closure under conditions of high CO_2_ and ABA [49]. Interestingly, *VviSLAC1* transcript abundance decreased in response to dehydration (Fig. 2). In contrast, the transcript abundance of *VviNRT*1 increased significantly in response to dehydration most notably in Cabernet Sauvignon and Ramsey leaves (Fig. 2). NRT1 acts as both a low- and high-affinity nitrate transporter in *Arabidopsis* depending on it phosphorylation status [50]. Nitrate concentrations appear to be important because nitric oxide (NO) is an important signaling molecule in ABA-induced stomatal closure and the production of NO is mediated through nitrate reductase activity [51].

Two additional transcripts shown to be within the ABA core-signaling network are the ABA-inducible HVA22-like homolog D (*HVA22D*) and mildew resistance locus 4 (*MLO4*). In our study, *VviHVA22D* transcript abundance increased significantly, most notably in Ramsey at 24 h of dehydration (Fig. 2). In contrast, *VviMLO4* transcript abundance significantly decreased in response to dehydration, again most notably in Ramsey starting at 4 h of dehydration and continuing throughout the experiment (Fig. 2).

### Metabolic transcripts within the ABA core-signaling network

Multiple transcripts within the ABA core-signaling network are known to be involved in metabolism. Changes in the expression of a number of these genes were observed (Fig. 2). Transcript abundance changes occurred more noticeably in Ramsey. For example, *VviGPX3* encodes a glutathione peroxidase; its transcript abundance significantly decreased more than 1.5 log_2_ fold at 24 h of dehydration in Ramsey (Fig. 2). In addition, *VviBCAT2* (branched-chain amino acid transaminase 2), *VviGAT1* (involved in amino acid transport), and *VviCAD5* (cinnamyl alcohol dehydrogenase) significantly increased in expression in response to dehydration (Additional files 3 and 6). The transcript abundance of an alcohol dehydrogenase, *VviADH1*, increased in response to dehydration within 4 h in Cabernet Sauvignon leaves with both Riparia Gloire and Ramsey increasing later in the experiment (Fig. 2).

### Kinase transcripts within the ABA core-signaling network

In addition to *VviOST1*, a number of other transcripts encoding kinases significantly changed in response to dehydration. For example, a number of SnRK3 kinases significantly increased in expression in response to dehydration (Fig. 2). These kinases are involved in a number of plant stress responses including cold, salt, and drought [52]. For example, the transcript abundance of *VviSnRK3.11* increased most notably in Riparia Gloire at 2 h of dehydration (Fig. 2). Another, *VviSnRk3.16* increased with a peak in expression at 8 h in both Cabernet Sauvignon and Ramsey with little response in Riparia Gloire. Finally, the transcript abundance of *VviSnRK3.6* increased in all genotypes surveyed, the earliest in Riparia Gloire leaves at 4 h of dehydration (Fig. 2).

Another kinase of note changing in response to dehydration is a diacylglycerol kinase (DGK), *VviDGK2* (Fig. 2). DGK synthesizes phosphatidic acid (PA), which is an important lipid-signaling molecule in plants involved in both biotic and abiotic signaling pathways [53]. The transcript abundance of *VviDGK2* increased as early as 1 h of dehydration in all genotypes (Fig. 2). The largest increase was observed in Cabernet Sauvignon leaves with a log_2_ fold increase of nearly 2.5, with Riparia Gloire lower at 1.8, and Ramsey at 1.2.

Other kinases increasing in expression include *VviETR2* and *VviMAP3Kδ4*. Ethylene receptor 2 (ETR2) is a member of a group of ethylene receptors, which upon binding ethylene initiate a large signaling cascade (see below). In response to dehydration, *VviETR2* increased in all genotypes with Cabernet Sauvignon and Ramsey increasing within 2 h of dehydration followed by Riparia Gloire at 4 h (Fig. 2). In contrast, *VviMAP3Kδ4* encodes an activated mitogen kinase, which increased in Riparia Gloire most notably at 2 h of dehydration followed by both Cabernet Sauvignon and Ramsey.

Two examples of kinases that significantly decreased in transcript abundance were a CRINKLY4 related 2 (*VviCCR2*) and *VviWNK2* (with no lysine (K)) (Fig. 2); *VviCCR2* decreased with a log_2_ fold change of −1.8 and −1.6 in Cabernet Sauvignon and Ramsey, respectively, with little response in Riparia Gloire. In contrast, *VviWNK2* displayed a log_2_ fold decrease of more than 2 in Riparia Gloire at 24 h of dehydration, the lowest observed (Additional file 3). Together, these data indicate significant changes in the expression of a number of kinases involved in multiple processes.

### Transcription factor transcripts within the ABA core-signaling network

There are many transcription factors in the ABA core-signaling network. An investigation into the expression of all is outside the scope of this work. Instead, interesting differences between the genotypes are highlighted. Transcription factors from multiple families are represented indicating changes in many different signaling cascades. For example, a number of transcripts from the MYB (myeloblastosis), NAC (for NAM (no apical meristem), ATAF (Arabidopsis transcription activation factor), CUC (cup-shaped cotyledon)), and AP2/ERF (APETALA2/Ethylene-Responsive Element Binding Protein) domain transcription factor families changed significantly between the genotypes in response to dehydration.

Two MYB genes in particular within the ABA core-signaling network responded differently to dehydration. The transcript abundance of *VviMYB12* decreased particularly in Riparia Gloire beginning at 1 h of dehydration (Fig. 2). In *Arabidopsis*, MYB12 regulates flavonoid biosynthetic genes [54] and subsequent reactive oxygen species (ROS) scavenging leading to greater drought tolerance [55]. Conversely, a MYB77-like gene, *VviMYB77* increased in expression particularly in Cabernet Sauvignon at 4 h of dehydration. MYB77 responds to ethylene and is involved in stress memory [56].

The transcript abundance of another two genes classified as homeodomain leucine zipper class I transcription factors, *VviHB6* and *VviHB12*, changed significantly between the genotypes in response to dehydration. *VviBH6* transcript abundance decreased in expression, particularly in Ramsey at 24 h of dehydration (Fig. 2). In contrast, *VviBH12,* followed a similar pattern in all genotypes displaying a decrease in expression early followed by an increase throughout the rest of the experiment.

One of the largest groups of transcription factors in plants is the AP2/ERF superfamily. With more than 130 members in *Vitis*, this particular family is known to regulate many different processes such as response to biotic and abiotic stress, development, reproduction, and response to hormones [57]. Recently, Cramer et al. [23] reanalyzed the phylogeny of this family in *Vitis*. Out of 130 family members on the *Vitis* microarray, 91 changed significantly in response to dehydration between the genotypes with 99 changing significantly in response to dehydration between genotypes over time (Additional files 3 and 6). This indicates that ethylene and ethylene signaling may play important roles in the dehydration response.

Recently, Dubois et al. [58] classified ERF6 and ERF5 as the “master regulators” of leaf growth in *Arabidopsis*. A number of ERF6-like transcription factors changed in response to dehydration. *VviERF6L3* and *VviERF6L1* responded similarly by increasing rapidly at 1 h of dehydration in Cabernet Sauvignon leaves with little response in both Ramsey and Riparia Gloire (Fig. 2). In contrast, the ERF/AP2 transcription factors, *VviRAP2.4* and *VviERF11*, displayed a similar pattern in Riparia Gloire increasing with a peak in expression at 2 h of dehydration. These data indicate a difference in the response between genotypes for multiple AP2/ERF transcription factors.

### Other hormone signaling transcripts within the ABA core-signaling network

Transcripts involved in ABA signaling are also known to interact with other hormone signaling pathways. For example, ARR5 (*Arabidopsis* response regulator 5) is an essential component of cytokinin signaling [59]. The transcript abundance of the closest *Vitis* ortholog, *VviARR5*, was decreased for all genotypes (Fig. 2). The transcript abundance in Cabernet Sauvignon decreased as early as 2 h of dehydration, however, at 24 h of dehydration the greatest changes were observed in Ramsey with a log_2_ fold change decrease of −2.0. The transcript abundance of *VviARR4* significantly decreased in response to dehydration (Fig. 2). This particular gene is known to be involved in the ethylene signaling pathway [60].

**Fig. 3:**
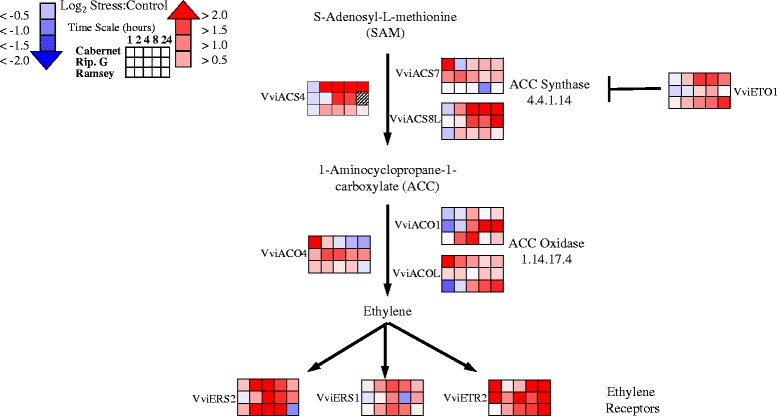
Transcript abundance of genes involved in ethylene metabolism and signaling. Corresponding *Vitis* loci ID and ANOVA results for the gene symbols used in this figure are listed in Additional file 6. Data are presented as heatmaps of mean values of a log_2_ ratio (Stress:Control), *n* = 3 at each time point

In plants, ethylene is synthesized from S-adenosine-L-methionine (SAM), and 1-aminocyclopropane-1-carboxylate (ACC). The conversion of SAM to ACC is catalyzed by ACC synthase (ACS), which is followed by the oxidation of ACC to ethylene catalyzed by ACC oxidase (ACO) [61]. ACS is the rate-limiting enzyme for ethylene biosynthesis. In our study, a number of putative ACS genes changed significantly in response to dehydration (Figs. 2 and 3). For example, transcript abundance of *VviACS2* increased in all genotypes, most notably in Riparia Gloire at 1 h of dehydration (Fig. 2). In both Riparia Gloire and Cabernet Sauvignon, gene expression remained high throughout the experiment while Ramsey was increased at 4 h of dehydration. Another example that has been linked to the ABA core-signaling network is *VviACS6* (Fig. 2). Interestingly, this gene was differentially regulated between the genotypes. The transcript abundance increased at 1 h in Cabernet Sauvignon, followed by Riparia Gloire, and the lowest expression was in Ramsey (Fig. 2).

Together, these data indicate that there are many changes in gene expression within the ABA core-signaling network as defined by Lumba et al. [42]. Changes in gene expression involved in processes such as transport, transcription factor expression, kinase expression, and hormone signaling, highlight a complex regulatory network that is involved in the response to dehydration.

### Dehydration induces significant changes in ethylene metabolism transcripts

Similar to ABA metabolism, massive changes in ethylene metabolism and signaling were observed. Within plants, small gene families encode multiple ACS and ACO genes that are known to be regulated differently depending on environmental, developmental, and hormonal signals [62–64]. In addition to the ACS genes previously mentioned, others changed significantly in response to dehydration (see Additional file 6 for gene annotations). *VviACS4* and *VviACS8*-like increased in expression in both Cabernet Sauvignon and Riparia Gloire with little response in Ramsey (Fig. 3). Transcript abundance of yet another, *VviACS7*, increased most notably in Cabernet Sauvignon leaves at 1 h of dehydration. Interestingly a negative regulator of ethylene production, ETHYLENE OVERPRODUCER 1 (*VviETO1*), also increased in expression, particularly in Cabernet Sauvignon and Ramsey (Fig. 3). Previously, Yoshida et al. [65] used yeast-two hybrid assays to establish that the *Arabidopsis* ETO1 interacts with AtACS5 and not other ACS proteins surveyed. There is no clear ortholog to AtACS5 in *Vitis;* therefore, further investigation is needed.

Downstream of ACS, transcripts for ACC oxidase also displayed a significant change in response to dehydration. In particular, both *VviACO4* and another ACO-like gene (*VviACOL*) increased in Cabernet Sauvignon leaves at 1 h of dehydration. *VviACO1* increased notably in Ramsey and 2 h and 8 h in Riparia Gloire with little response in Cabernet Sauvignon (Fig. 3).

### Dehydration induces significant changes in ethylene signaling transcripts

**Fig. 4:**
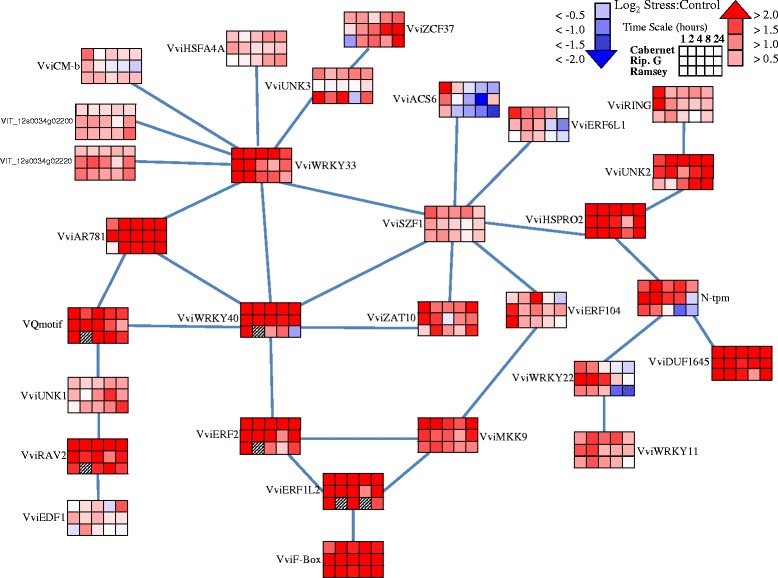
Representation of transcript abundance of some of the genes of the ethylene signaling network generated from ATTED-II database (see manuscript). Corresponding *Vitis* loci ID and ANOVA results for the gene symbols used in this figure are listed in Additional file 6. Data are presented as heatmaps of mean values of a log_2_ ratio (Stress:Control), *n* = 3 at each time point

A large number of transcripts involved in ethylene signaling significantly changed in response to dehydration (Figs. 3 and 4). The transcript abundance of a number of ethylene receptors increased significantly in response to dehydration (Fig. 3). Ethylene receptors are broken down into two subfamilies based on conserved histidine kinase domains and are localized within the endoplasmic reticulum (for review see [66]). Ethylene receptor 2 (*VviETR2*) increased notably in Cabernet Sauvignon and Riparia Gloire at 1 h of dehydration with Ramsey responding later during the treatment. Ethylene response sensor 2 (*VviERS2*) and *VviERS1* followed a similar pattern by increasing at 2 h of dehydration in Cabernet Sauvignon and Ramsey with a later response in Riparia Gloire (Fig. 3).

Additional transcripts involved in ethylene signaling were mapped (Fig. 4) using the ATTED-II database as a template [67]. The closest *Vitis* orthologs were determined according to Gramene ([43] release 44 (January 2015)). A detailed investigation of all transcripts within this ethylene-signaling network is outside the scope of this study, however, a few key genes are discussed here.

The transcript abundance of a number of WRKY domain transcription factors involved in ethylene signaling increased in response to dehydration (Fig. 4). The transcript abundance of *VviWRKY33* and *VviWRKY40* increased rapidly within 1 h of dehydration in all genotypes. The transcript abundance of *VviWRKY22* also increased rapidly within 1 h of dehydration in Riparia Gloire, but decreased at later time points in Ramsey indicating differences in the regulation of this transcription factor (Fig. 4). Recently, WRKY33 has been shown to bind directly to the promoter of ACS2 and ACS6 to induce gene expression in *Arabidopsis* [68]. AP2/ERF transcription factors appear to regulate the expression of WRKY40 [69], further indicating cross-talk between the WRKY transcription factors and ethylene signaling.

Within this network, AP2/ERF transcription factors were also observed to change significantly. For example, the transcript abundance of *VviERF6L1* increased at 1 h of dehydration with little response in Riparia Gloire and Ramsey (Fig. 4). The transcript abundance of *VviERF104* also increased at 1 h of dehydration, however, this increase was observed in Riparia Gloire and Ramsey with little response in Cabernet Sauvignon. Finally, The transcript abundance of *VviERF2* and *VviERF1L2* increased at 1 h of dehydration and remained high throughout the experiment, notably in Cabernet Sauvignon and Riparia Gloire leaves.

### Notable AP2/ERF transcript responses in drought sensitive Riparia Gloire

**Fig. 5:**
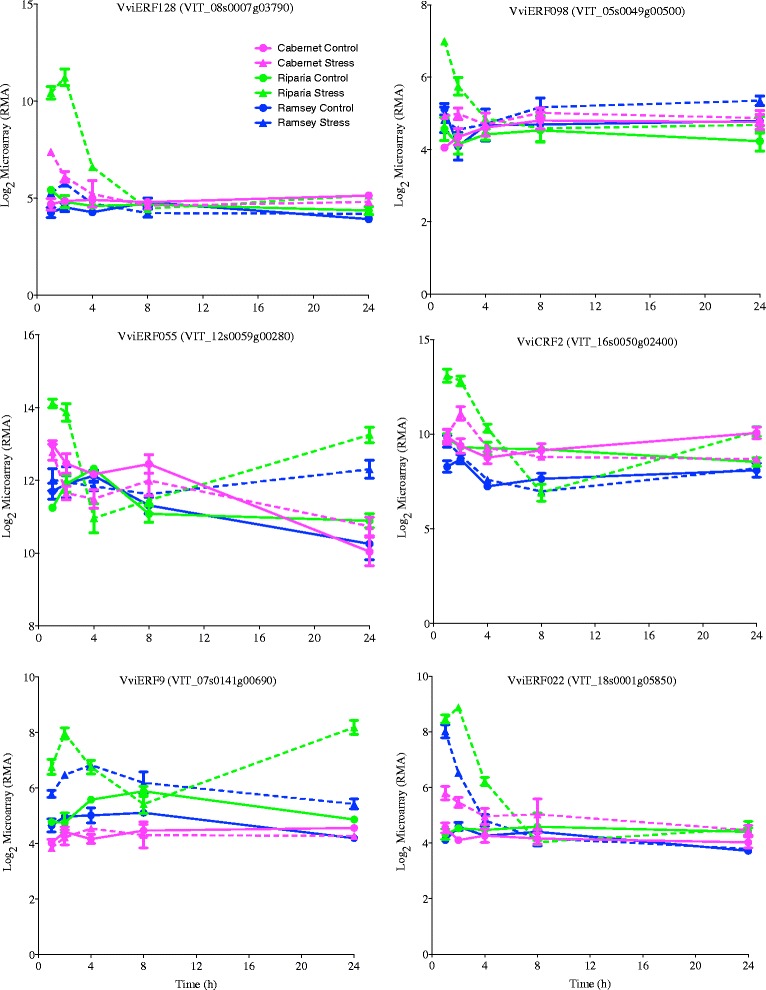
Transcript abundance of notable AP2/ERF transcripts in Riparia Gloire leaves. Corresponding *Vitis* loci ID and ANOVA results for the gene symbols used in this figure are listed in Additional file 6. Expression values are presented as the mean ± SE log_2_ values, *n* = 3 at each time point

Riparia Gloire leaves are more sensitive to rapid dehydration by closing their stomata more quickly compared to both Cabernet Sauvignon and Ramsey [3]. In this study, multiple AP2/ERF transcription factors increased in transcript abundance at 1 h in Riparia Gloire with little or no response in the other genotypes. For example, the transcript abundance of *VviERF128* increased rapidly at 1 h with a slight response in Cabernet Sauvignon and little response in Ramsey leaves (Fig. 5). According to Cramer et al. [23] this gene does not have a clear ortholog to *Arabidopsis* indicating the possibility for a unique function in *Vitis*. Another AP2/ERF transcript that had a similar pattern of expression was *VviERF098* (Fig. 5). The closest *Arabidopsis* ortholog *AtERF98* (At3g23230), increases ascorbic acid (AsA) biosynthesis leading to an increase in salt tolerance [70]. AsA has a number of roles in plants including as an antioxidant, protecting the plant from reactive oxygen species (ROS), which can result in enhanced tolerance to a variety of abiotic stresses [71, 72].

The transcript abundance of other AP2/ERF transcription factors in Riparia Gloire that increased rapidly in response to dehydration included *VviERF055* and *VviERF022* (Fig. 5). Interestingly, *VviERF055* is closely related to TRANSLUCENT GREEN (TG), an ERF family transcription factor in *Arabidopsis*. TG binds directly to the promoter of multiple aquaporin genes, and overexpression results in increased drought tolerance [73]. CYTOKININ RESPONSE FACTOR 2 (*VviCRF2*) is another example of an AP2/ERF transcript that increases in abundance in Riparia Gloire leaves with little to no response in the other genotypes (Fig. 5). The closest *Arabidopsis* ortholog, cytokinin response factor 2 (CRF2, At4g23750), interacts with other CRF proteins within the cytokinin signaling pathway [74].

The transcript abundance of two negative regulators of ethylene signaling, *VviERF9* and *VviERF022*, also increased rapidly at 1 h of dehydration in Riparia Gloire leaves. *VviERF9* belongs to subgroup VIII in the ERF family [23, 75], which is known to be involved in transcriptional repression [76–78]. In our study, *VviERF9* expression increased rapidly in Riparia Gloire, to a lesser extent in Ramsey, with no response in Cabernet Sauvignon (Fig. 5). *VviERF022* in Riparia Gloire and Ramsey leaves increased in expression at 1 h of dehydration, followed by a decrease at later time points. Little is known about the function of this gene in *Vitis*. Nowak et al. [79] demonstrated the importance of AtERF022, the closest ortholog, in somatic embryogenesis in *Arabidopsis*. This study provides direct evidence that ERF022 regulates the biosynthesis and signaling of ethylene. These data indicate some unique changes occurred in Riparia Gloire leaves within the AP2/ERF family. Changes in both positive and negative regulators of ethylene signaling also indicate a complex regulatory network requiring further investigation.

Together, these data provide further evidence for changes in the expression of genes involved in both ABA and ethylene metabolism and signaling in response to dehydration. These changes were also observed to be different between the genotypes surveyed indicating different types of transcriptional regulation.

### Network analysis: WGCNA

**Fig. 6:**
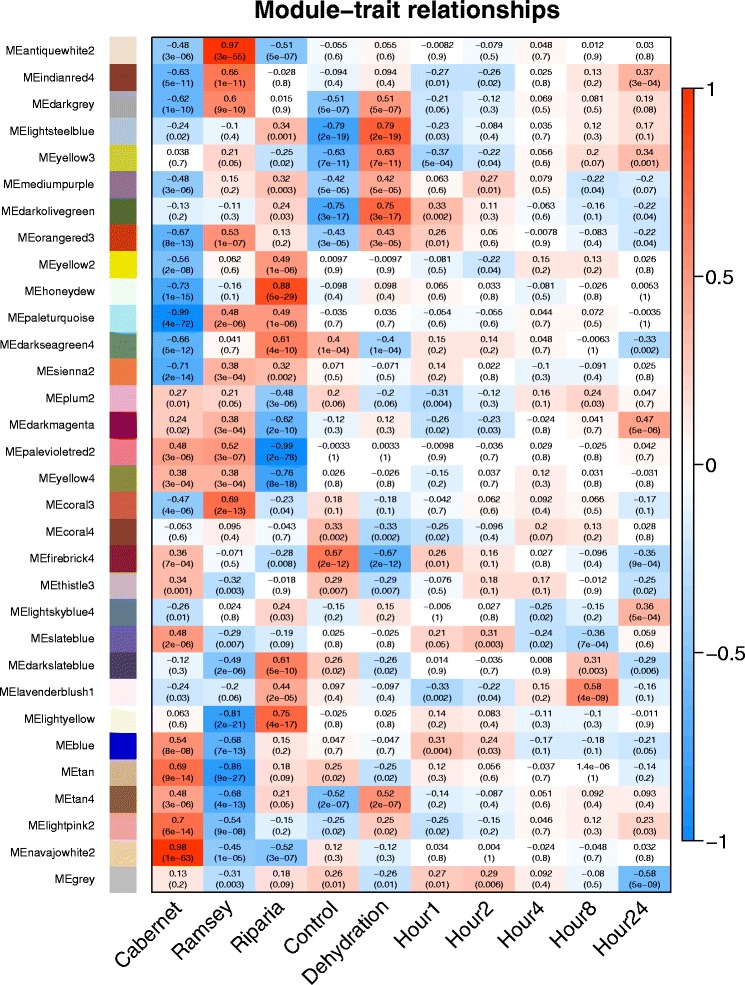
Heatmap of the correlation of WGCNA modules with treatments (traits)

Weighted gene coexpression network analysis (WGCNA) was performed to get a better understanding of which genes within these complex signaling networks were the most connected hubs. Thirty modules or gene networks were detected, assigned color names, and correlated to genotype and treatment effects over time (Fig. 6). All genes were correlated with these 30 colored modules; the grey category is not a true module, but a place to put all the leftover genes not correlated well enough with one of the significant colored modules. In addition, a measure called the kME (module eigengene-based connectivity) was calculated for each gene to every module (Additional file 7). Genes with a kME score of 1 are perfectly positively correlated with that particular module (network) and scores of −1 are perfectly negatively correlated to the module. The advantage of computing kME scores for each module is that genes can act as a hub in more than one module or network. Genes with the highest kME score have the most connectivity and are therefore designated the largest hubs. The largest hubs were identified along with the top GO categories overrepresented in each module (Additional file 8). All top hub genes had kME scores of 0.92 or higher. Some of the top hub genes in each module were of unknown function, making them interesting candidates for future research (Additional files 7 and 8).

In contrast to previous GO enrichment analyses done with Genotype x Treatment or the Genotype x Treatment x Time subsets of genes, GO enrichment analysis of the individual modules identified with WGCNA provided much more significant results with more biological meaning. Some of the modules were highly enriched (based upon their *p*-value) in gene ontologies such as plum2 (translation), firebrick4 (photosynthesis) and yellow2 (protein amino acid phosphorylation); a few were less distinct or not significant, such as yellow3, blue, and tan4 (Additional file 8).

**Fig. 7:**
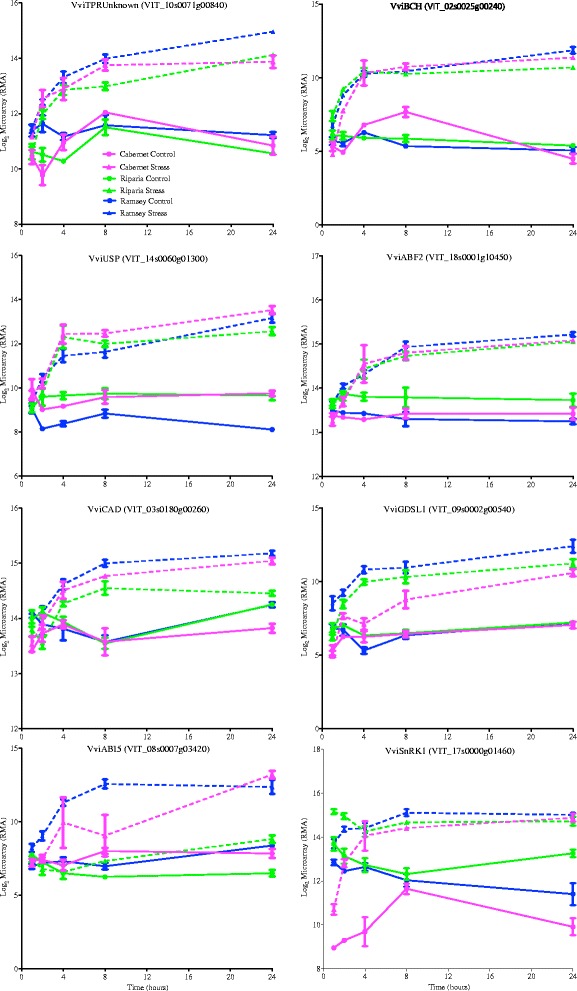
Transcript abundance of some of the top gene hubs in the yellow3 module. Expression values are presented as the mean ± SE of the log_2_ values, *n* = 3 at each time point

The most interesting modules were modules (lightsteelblue, darkolivegreen, orangered3, and yellow3) because they were correlated positively with dehydration and genotype (Fig. 6). The most interesting module is the yellow3 module, because the heat map for genotypes was most highly correlated with the genotype order of drought tolerance: Ramsey > Cabernet Sauvignon > Riparia Gloire, and there was an increase in response to dehydration. The top hub gene in the yellow3 module was a gene for an unknown protein with a TPR domain (Additional file 8; Fig. 7). TPR domains are structural motifs that facilitate protein-protein interactions and assembly of protein complexes. There were several other TPR domain protein genes in the top of this module. These genes are clear targets for future research due to lack of knowledge of these genes in any plant species.

The yellow3 module is overrepresented with a significant but relatively high *p*-value (1.28 × 10^−4^) in gas (oxygen) transport. Basically this means that either this network of genes is poorly annotated or there is a general mix of all kinds of gene functions that is not very distinct from the entire genome. Nevertheless, there is a very interesting subset of hub genes all having kME values above 0.80, including the before mentioned ABA core-signaling transcription factors, ABF2 and ABI5 (Fig. 7; Additional file 7). Other notable hub genes in this module are a SNF1-related protein kinase (*VviSnRK1*; a central regulator of metabolism), a cinnamyl alcohol dehydrogenase (*VviCAD*; phenylpropanoid metabolism), a β-carotene hydroxylase (*VviBCH*; carotenoid metabolism), *GSDL Lipase 1* (*VviGLIP1*; ethylene signaling) and a universal stress protein (*VviUSP*; defense). The one gene that stands out in this group is *VviABI5*, because of the differences displayed in transcript abundance between the genotypes in their response to dehydration with Ramsey > Cabernet Sauvignon > Riparia Gloire. There are 81 hub genes in this network with a kME higher than 0.80, indicating a very complex network.

**Fig. 8:**
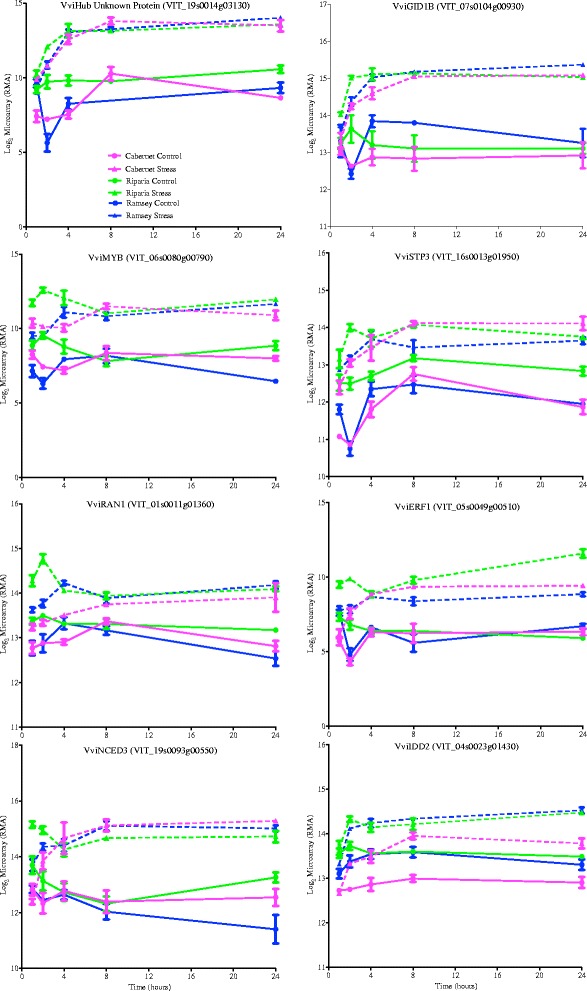
Transcript abundance of some of the top gene hubs in the lightsteelblue module. Expression values are presented as the mean ± SE log_2_ values, *n* = 3 at each time point

The other very interesting module is the lightsteelblue module (Additional file 7). A number of the genes with high kMEs in the yellow3 module also have high kMEs in the lightsteelblue module, including *VviABI5*, *VviABF2*, *VviSnRK1*, some wound-inducible genes, and genes encoding late-embryogenesis abundant (LEA) proteins or dehydrins. This module is significantly enriched in the ethylene signaling GO category. The top hub gene is an unknown gene, whose ortholog in *Arabidopsis* is induced by Al (Fig. 8; Additional files 7 and 8). Other top hub genes with similar but slightly different expression patterns are Gibberellin Insensitive Dwarf 1B (*VviGID1B*; a gibberellin receptor), an uncharacterized *VviMYB*, RAS-related Nuclear Protein (*VviRAN1;* GTPase signaling), *VviERF1* (ethylene signaling), *VviNCED3* (ABA biosynthesis), and Indeterminate Domain 2 (*VviIDD2;* a C2H2-type zinc finger protein). Interestingly, *VviERF1*, which is at the start of the ERF transcription factor cascade is more highly expressed in Riparia Gloire in response to dehydration (Fig. 8). Thus there is a clear overlap in ABA and ethylene signaling hubs in these two modules, which supports the argument that there is strong crosstalk between these two hormone signaling pathways.

Top hub genes from other modules with high GO enrichment categories such as translation, photosynthesis, phenylpropanoid metabolism were less interesting because the transcripts in these sets were less correlated with drought tolerance, and therefore will not be discussed further. We have only just scratched the surface of the analyses of these data. This dataset (Additional file 7) is very deep and can be exploited for years to come. Many other fascinating networks are present within this dataset but there is not enough space and time to describe them here.

## Discussion

There are thousands of different grapevine genotypes leading to a large pool of natural genetic diversity [80, 81]. The phylloxera devastation of the European wine industry during the 19^th^ century resulted in the introduction of North American genotypes as rootstocks for biotic and abiotic stress resistance [6]. With global climate change, a better understanding of the genes involved in drought tolerance will be needed. Previous work by Padgett-Johnson et al. [12] clearly showed differences in water-use-efficiency between different genotypes of grapevine in the field. However, studies like this require multiple years for the establishment of an experimental vineyard.

### The strengths and weaknesses of the rapid dehydration assay

To complement previous field studies, Hopper et al. [3] developed a rapid dehydration assay that is amenable to large-scale phenotyping studies. The assay takes only a few hours and can precisely control the level of dehydration. It is simple, not requiring expensive or sophisticated equipment. It can be done at any time of the year as long as plant material is available, such as from a greenhouse. The results from the rapid dehydration assay are consistent with field assays, indicating that it is a suitable assay for evaluating dehydration tolerance. This assay is excellent as a first screen for genetic tendencies.

The screen only reflects the inherent tendencies of a leaf. The leaf is isolated from the rest of the plant and cannot sense signals from other parts of the plant. For example, in an earlier study, massive changes in proteins were detected in Cabernet Sauvignon shoots that had been gradually dehydrated [82]. These changes occurred before any photosynthetic or growth symptoms indicating that the shoot detected a feed-forward signal from another part of the plant, such as the roots.

This rapid dehydration assay by its nature cannot look at the more gradual changes that occur in a plant in a more natural setting. Nevertheless, it was able to detect differences between genotypes that are reflective of their behavior in the field.

### Sensitivity and timing of stress responses

One of the strengths of our transcriptomic approach in this study was the collection of samples along a detailed time course. This allowed detection of genotype sensitivity to dehydration. Sensitivity could reflect sensitivity to injury and thus indicate a stress-sensitive (dehydration sensitive) plant or it could indicate a plant with heightened awareness of impending drought, giving it more time to respond and thus fostering dehydration resistance. The use of genotypes differing in their drought tolerance allowed us to distinguish between these sensitivities. ABA signaling appears to be key in this response with the drought tolerant genotype, Ramsey, showing heightened responses in ABA signaling and decreased responses in ethylene signaling relative to the drought sensitive genotype, Riparia Gloire, which showed slower responses in ABA signaling and heightened responses in ethylene signaling. The intermediate genotype, Cabernet Sauvignon, was consistent with these responses, being intermediate in both ABA and ethylene signaling.

### The importance of ABA and ethylene responses to dehydration

ABA is a major plant hormone involved in plant responses to dehydration. Significant differences in transcript abundance of genes involved in ABA metabolism and signaling were observed between the genotypes. Previously, Hopper et al. [3] found that application of ABA to leaves prior to dehydration decreased the overall amount and rate of water lost. Differences between the genotypes in their stomatal conductance after application at various concentrations of ABA also indicate differences in ABA sensitivity. Differences observed in water loss are presumably through differences in stomatal conductance and regulation of gene transcription, protein synthesis and other signaling pathways (for review see [83]).

Based on both the standard and network analysis approaches, significant changes in the transcript abundance of genes involved in ABA metabolism and signaling were observed (Figs. 2 and 3). For example, the transcript abundance of the three *NCED* genes in *Vitis* that catalyze the rate-limiting step in ABA biosynthesis increased in response to dehydration. Interestingly, *VviNCED6*, is increased in both Riparia Gloire and Ramsey with a different response in Cabernet Sauvignon. This indicates differences in the regulation of these key genes to dehydration. Recently, Lumba et al. [42] created an ABA interactome using a systems biology approach. Utilizing transcriptomic and proteomic data, these authors were able to create a detailed interactome with over 500 interactions, highlighting the complexity of ABA signaling. Studies like this also highlight the need and utility for transcriptomic data such as in the study presented here. Our study provides further evidence for differences in ABA metabolism and signaling at the transcript level that may be involved in dehydration adjustment for the *Vitis* genotypes.

Key candidate factors appear to be the transcription factors, ABI5 and ABF2, at the start of the ABA transcriptional signaling pathway. In particular, the transcriptional response of *VviABI5* to dehydration in Ramsey was more sensitive than Cabernet Sauvignon, which was more sensitive than Riparia Gloire. This makes it a good target for future research.

*ABI5* was first cloned in *Arabidopsis* [84]. Its expression is highest in seeds, but a low level of expression is observed in vegetative tissues. The expression of some *AtLEA* genes is dependent upon *AtABI5* expression in *Arabidopsis*. Note that there were several *VviLEA* genes within the top kMEs (>0.80) in the yellow3 module (Additional file 7). *ABI5* expression is largely associated with seed development and not vegetative organs or tissues [85]. However recent evidence has linked *ABI5* to vegetative functions such as the induction of flowering [86], leaf senescence [87], drought tolerance [88], and stomatal movement [89]. With these exciting new findings, it is necessary to focus more research on the function of *ABI5* in grapevine vegetative organs.

The SnRK1 γ-subunit, another hub in the yellow3 module is a part of the SnRK1 complex that is a central regulator of metabolism. SnRK1 responds to sugar and ABA signaling [90]. PP2Cs (ABI1 and AHG3) directly dephosphorylate the SnRK1 α-subunit [91]; thus, the inhibition of PP2C activity by ABA directly stimulates SnRK1 expression and activity, complementing the stress response through energy regulation and coordination.

Ethylene is another plant hormone involved in dehydration stress signaling (for review see [76]). The ethylene response may reflect dehydration adjustment, damage control or senescence. The ERF domain of ERF transcription factors contains 60 to 70 conserved amino acids and was first identified in four DNA binding proteins NtERF1-4 from *Nicotiana tabacum* [92]. Group I ERF transcription factors have previously been shown to be important in mediating drought tolerance in plants [93–95]. *VviERF1* had a high kME in the lightsteelblue module, which was significantly enriched in ethylene signaling gene ontologies. The closest orthologs to *VviRAP2.4* and *VviERF055* belong to Group I in *Arabidopsis* [23] and were both up-regulated in response to dehydration, most notably in Riparia Gloire leaves (Figs. 2 and 5).

ERF5 and ERF6 are also important transcription factors involved in drought signaling and have been called the “master regulators” in leaf growth in response to environmental changes [58]. In Cabernet Sauvignon leaves, a number of *VviERF6-like* transcription factors increased in response to dehydration (Fig. 2). A similar trend in the expression of *VviERF6-lik*e genes was seen as sugar (°Brix) levels increased within the skins of ripening Cabernet Sauvignon berries [23]. This may indicate a similar level of regulation of these genes in response to dehydration, ripening or other stress responses.

Similar to ABA, transcripts involved in ethylene metabolism and signaling also changed significantly in response to dehydration (Figs. 3 and 4). A number of transcripts were observed to be changing significantly based on the Genotype **x** Treatment and Genotype **x** Treatment **x** Time interaction terms indicating a different response between the genotypes in response to dehydration and over time.

There is substantial evidence that there is crosstalk between ABA and ethylene in response to dehydration [88, 96]. Overexpression of an ethylene responsive transcription factor RAV2 along with the ABA-response transcription factor ABI5 had synergistic effects on drought tolerance in cotton [88]. Interestingly, *VviRAV2* has a high kME (0.81) in the lightsteelblue module along with *VviABI5* (Additional file 7). Lumba et al. [42] provide evidence for hormonal crosstalk with a number of genes involved in ethylene metabolism and signaling within the ABA core network. For example, ACS catalyzes the rate-limiting step in ethylene biosynthesis and ACS6 in *Arabidopsis* is regulated by ABA Insensitive 1 (ABI1), a negative regulator of ABA signaling [97]. Multiple ACS genes in *Vitis* changed significantly between the genotypes in response to dehydration (Figs. 2 and 3).

## Conclusion

In summary, the leaf dehydration time-series assay allowed the detection of a very large number of transcriptional changes in a coordinated fashion. Key genes were identified by a standard *a posteriori* analysis that involved mapping known biochemical pathways in leaves responding to dehydration (e.g. ABA and ethylene metabolism and signaling pathways). An *a priori* data analysis approach using WGCNA proved more powerful; it confirmed the results from the standard approach and it identified 30 distinct modules (networks), most of which had highly enriched GO categories that were biologically and functionally relevant. In addition, WGCNA enabled the identification of gene hubs in these modules that are likely to be important and significant operators within these networks.

The results from this study confirmed our hypothesis that ABA signaling pathways were different between the grapevine genotypes. Furthermore, the results indicate that the dehydration response had substantial cross-talk between the ABA and ethylene signaling pathways. Some of the genes in the ABA and ethylene signaling pathways were highly connected hubs and were correlated with drought tolerance. A number of interesting unknown genes were also identified and associated with these pathways. *VviABI5* is one of the best candidate hubs for drought tolerance because its expression was more rapid and higher in the drought tolerance genotypes and because of its known functions in ABA signaling and drought tolerance. The facts that this gene is normally associated with seed dormancy, that the transcript abundance of *VviABI5* in leaves increased significantly in a species (*Vitis champinii*) that has evolved in a hot and dry climate, and did not respond substantially in the leaves of a species (*Vitis riparia*) that has evolved in a cooler and wetter climate, make this gene particularly interesting.

This study provides a very rich data set that can be further explored for new discoveries. Only the tip of this data “iceberg” has been discussed here. Future research will focus on the further elucidation of the regulation of these gene networks and on gradual responses of the root to dehydration, since multiple lines of evidence indicate it may act as the initial sensor for drought stress signaling.

## Additional files

Additional file 1: Principal component analysis (PCA) of expression data. Symbol names refer to genotype (Ram = Ramsey, Rip = Riparia and CS = Cabernet Sauvignon); the following number is the time in hours (1, 2, 4, 8, and 24), then there is a separating “.”, followed by the treatment (C = control, S = stress or dehydration), and the final number refers to the sample replicate number. (PDF 19 kb) Additional file 2: WGCNA sample dendrogram and trait heatmap of expression data. (PDF 29 kb) Additional file 3: Annotation, transcript abundance values, and statistics of all genes on the NimbleGen Grape Whole-Genome microarray. (XLSX 20763 kb) Additional file 4: Overrepresented GO categories of all significantly changing transcripts in response to Genotype x Treatment interaction. (XLSX 192 kb) Additional file 5: Overrepresented GO Categories of all significantly changing transcripts with the Genotype x Treatment x Time interaction. (XLSX 130 kb) Additional file 6: Updated annotation of genes involved in ABA metabolism and signaling, ethylene metabolism and signaling, and transcription factors including those in Figs. 1, 2, 3 and 4. (XLS 285 kb) Additional file 7: kME values of all transcripts in each of the gene modules determined by WGCNA. (XLSX 15417 kb) Additional file 8: Summary table of WGCNA modules including top hub genes and gene ontologies. (XLSX 50 kb)
