# Supplementary figures and images for: Transcriptomic network analyses of leaf dehydration responses identify highly connected ABA and ethylene signaling hubs in three grapevine species differing in drought tolerance
Source: BMC Plant Biol. 2016 May 23;16:118. doi: 10.1186/s12870-016-0804-6 (PMC4877820; doi:10.1186/s12870-016-0804-6)

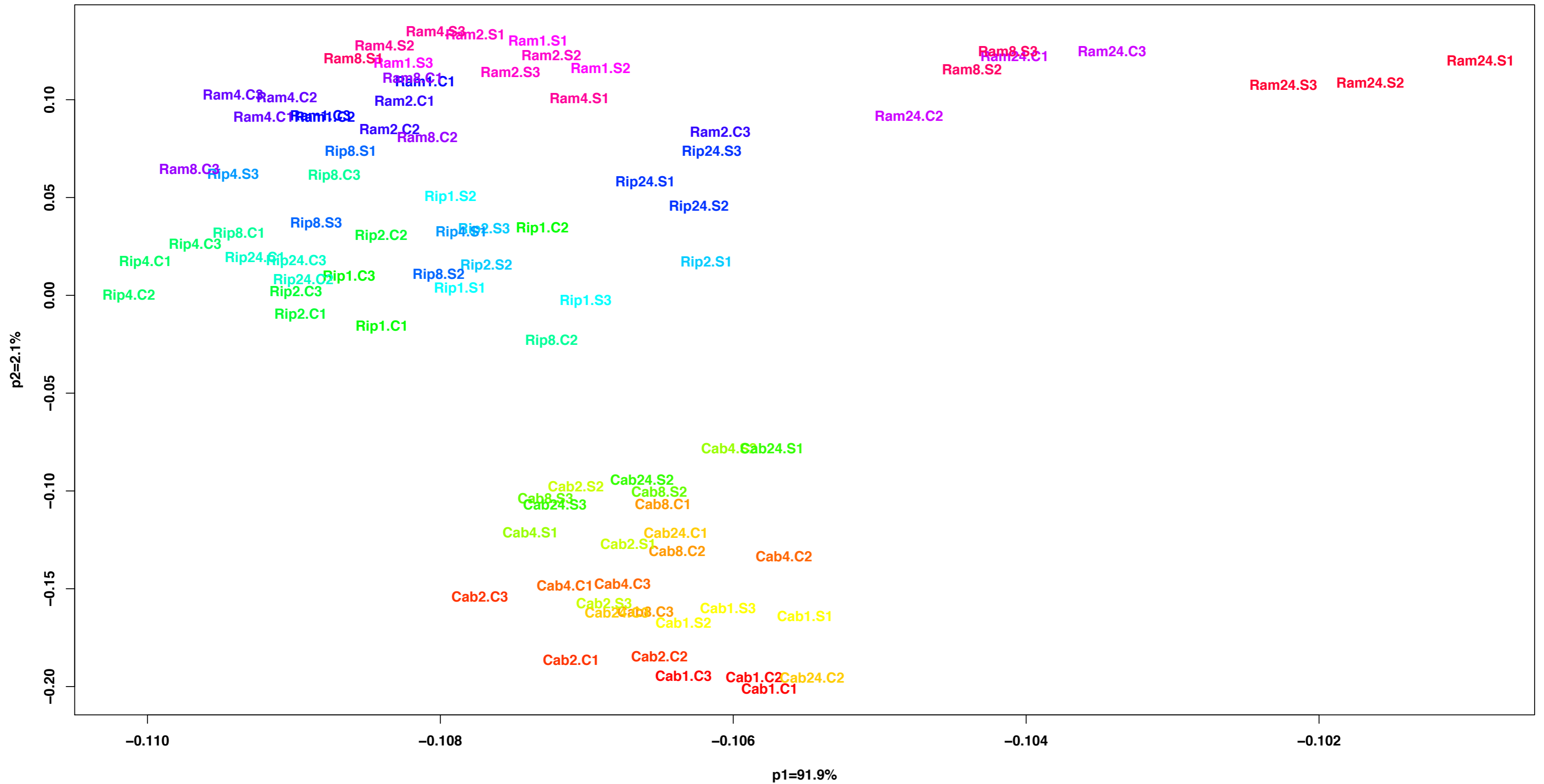

Supplement: Additional file 1: — Principal component analysis (PCA) of expression data. Symbol names refer to genotype (Ram = Ramsey, Rip = Riparia and CS = Cabernet Sauvignon); the following number is the time in hours (1, 2, 4, 8, and 24), then there is a separating “.”, followed by the treatment (C = control, S = stress or dehydration), and the final number refers to the sample replicate number. (PDF 19 kb) [file 12870_2016_804_MOESM1_ESM.pdf]

## Sample dendrogram and trait heatmap

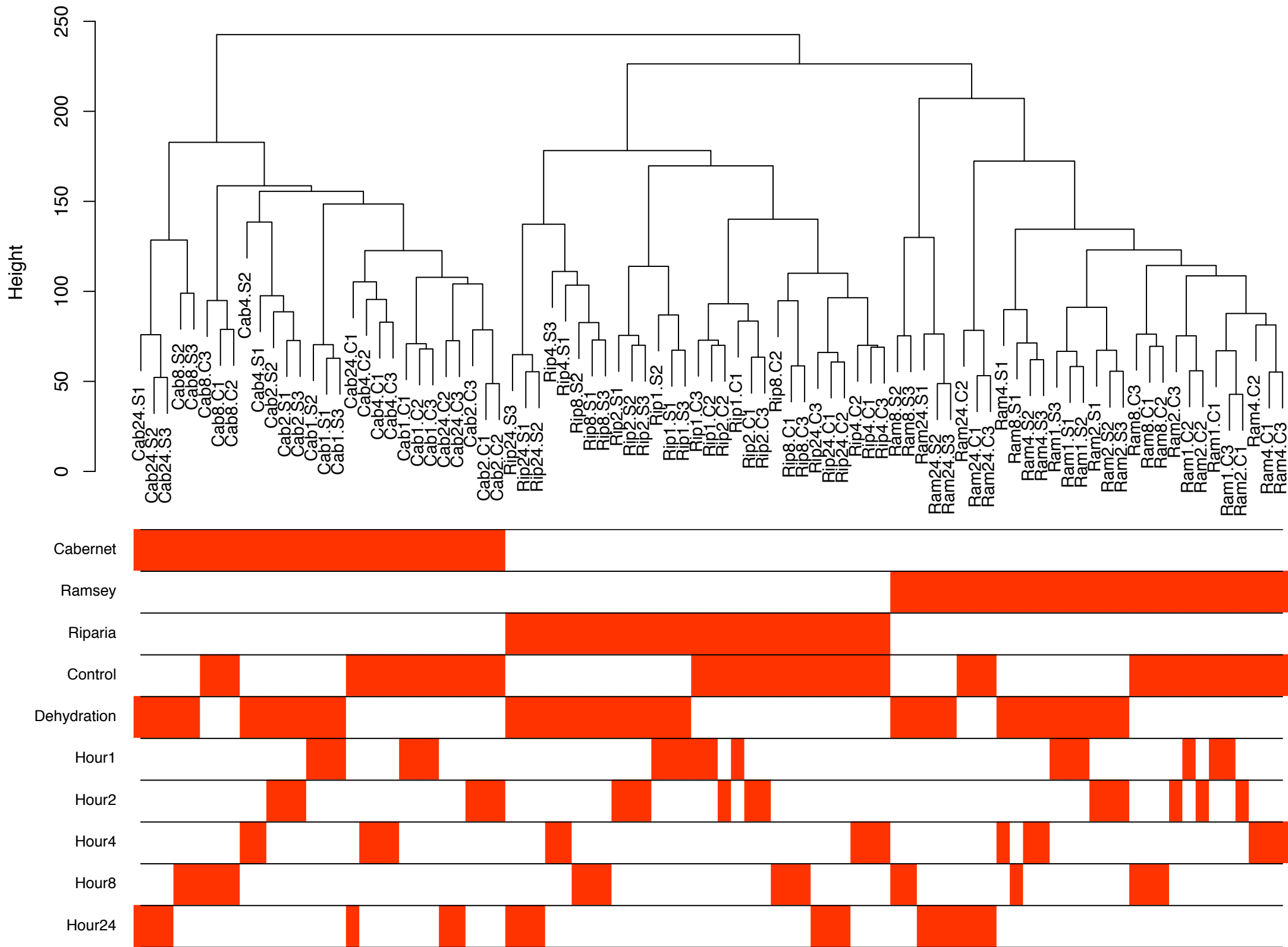

Supplement: Additional file 2: — WGCNA sample dendrogram and trait heatmap of expression data. (PDF 29 kb) [file 12870_2016_804_MOESM2_ESM.pdf]
